# Caspase-2 Short Isoform Interacts with Membrane-Associated Cytoskeleton Proteins to Inhibit Apoptosis

**DOI:** 10.1371/journal.pone.0067033

**Published:** 2013-07-01

**Authors:** Chunhua Han, Ran Zhao, John Kroger, Meihua Qu, Altaf A. Wani, Qi-En Wang

**Affiliations:** 1 Department of Radiology, The Ohio State University Wexner Medical Center, Columbus, Ohio, United States of America; 2 Department of Pharmacology, Weifang Medical University, Weifang, China; 3 Comprehensive Cancer Center, The Ohio State University, Columbus, Ohio, United States of America; Innsbruck Medical University, Austria

## Abstract

Caspase-2 (casp-2) is the most conserved caspase across species, and is one of the initiator caspases activated by various stimuli. The *casp-2* gene produces several alternative splicing isoforms. It is believed that the long isoform, casp-2L, promotes apoptosis, whereas the short isoform, casp-2S, inhibits apoptosis. The actual effect of casp-2S on apoptosis is still controversial, however, and the underlying mechanism for casp-2S-mediated apoptosis inhibition is unclear. Here, we analyzed the effects of casp-2S on DNA damage induced apoptosis through “gain-of-function” and “loss-of-function” strategies in ovarian cancer cell lines. We clearly demonstrated that the over-expression of casp-2S inhibited, and the knockdown of casp-2S promoted, the cisplatin-induced apoptosis of ovarian cancer cells. To explore the mechanism by which casp-2S mediates apoptosis inhibition, we analyzed the proteins which interact with casp-2S in cells by using immunoprecipitation (IP) and mass spectrometry. We have identified two cytoskeleton proteins, Fodrin and α-Actinin 4, which interact with FLAG-tagged casp-2S in HeLa cells and confirmed this interaction through reciprocal IP. We further demonstrated that casp-2S (i) is responsible for inhibiting DNA damage-induced cytoplasmic Fodrin cleavage independent of cellular p53 status, and (ii) prevents cisplatin-induced membrane blebbing. Taken together, our data suggests that casp-2S affects cellular apoptosis through its interaction with membrane-associated cytoskeletal Fodrin protein.

## Introduction

Apoptosis is a highly conserved mechanism which plays an important role in normal development and tissue homeostasis [1]. Apoptosis is also one of the cell death mechanisms that can be triggered in cancer cells by various cancer treatment schemes, e.g., chemotherapy, radiotherapy, immunotherapy or targeted therapy [2], [3], [4], [5]. Apoptosis usually requires the activation of a series of cysteine aspartate-specific proteases referred to as caspases [6]. Caspases, whose activation is a hallmark of apoptosis, are a family of proteins that are one of the main effectors of apoptosis. To date, about 14 mammalian caspases have been identified, and can be classified into three groups based on their function: inflammatory caspases, apoptotic initiator caspases, and apoptotic effector caspases (Reviewed in [7]).

Capase-2 is the most conserved caspase across species [8], [9]. Despite its early discovery, caspase-2's physiological function has long remained an enigma [10]. The difficulty in determining its function is due to the existence of two caspase-2 isoforms, each serving opposing functions in apoptosis. The caspase-2 gene produces several alternative splicing isoforms. The inclusion of exon 9 incorporates an in-frame stop codon in the casp-2 short isoform (casp-2S) mRNA, producing a truncated protein that inhibits cell death. The exclusion of exon 9 results in the casp-2 long isoform (casp-2L) mRNA, whose protein product induces cell death [8], [11]. However, further characterization of casp-2S isoform (Nedd2S) indicated that casp-2S did not act as a general inhibitor of apoptosis in all cell types and it did not exert its effect by directly competing with casp-2L [12]. The levels of casp-2L and casp-2S are governed by alternative promoters and splicing [13]. The average casp-2L/2S mRNA ratio is always high, and is often above 100-fold in several cell lines including leukemia (U937), carcinoma (HeLa, HCT116, HepG2, HT29), and immortalized (293T) [14] cells.

The subtle phenotype of casp-2 knockout mice does not clarify the biological role of this protein because both casp-2L and casp-2S are deficient in the mice (Reviewed in [15]). Similarly, confounding data was also generated in siRNA-based studies. Casp-2 downregulation by siRNA was initially reported to strongly inhibit etoposide-induced cell death [16], but doubts have been raised regarding the specificity of the siRNA used in these experiments [17]. The lack of siRNA specific to the distinct isoforms of casp-2 also makes this data open to questioning. Other researchers observed weak to no protection against etoposide-induced apoptosis, using a variety of techniques to abolish casp-2 expression in a number of cell types [10], [18], [19], [20], [21].

Apoptosis is characterized by cell membrane blebbing, cell shrinkage, chromatin condensation, and DNA fragmentation [22]. Using casp-2S overexpression, Droin et al [23] demonstrated that casp-2S selectively inhibits chromatin condensation, apoptotic body formation, membrane blebbing, and phosphatidylserine externalization following etoposide treatment in the human leukemic cell line U937. Regarding the mechanisms underlying the anti-apoptotic role of Casp-2S, it has been reported that casp-2S antagonizes apoptosis by inhibiting the activation of casp-2L and caspase-3, preventing ROCK-1-mediated apoptotic blebbing and body formation [24], [25]. However, simple overexpression in cells is not optimal for studying the physiological function of this protein. By using casp-2S specific siRNA, we have recently demonstrated that the downregulation of casp-2S enhanced the cleavage of PARP, the activation of caspase-9 and caspase-6, and the phosphatidylserine externalization in XPC-deficient human fibroblasts [21].

Fodrin (αII-Spectrin) and the Fodrin-based cytoskeleton confer resiliency and durability to integral membrane proteins, and are believed to participate in the formation and maintenance of specialized membrane subdomains [26]. The cleavage of Fodrin leads to the disruption of the actin filament network. This disruption may specifically contribute to the loss of overall cell shape and detachment from the matrix during apoptosis [27]. Fodrin cleavage is thought to be involved in the membrane blebbing observed during apoptosis [28]. Fodrin is susceptible to cleavage by both calpain and caspases. Fodrin cleavage by calpain is thought to be involved in membrane remodeling, necrosis, and apoptosis. The cleavage of Fodrin induced by caspases appears to occur only during apoptosis [29].

In this study, we assessed the role of casp-2S in cancer cells during apoptosis through gain-of-function and loss-of-function strategies. Our studies using casp-2S-specific siRNA clearly demonstrate the anti-apoptotic role of casp-2S. In addition, we have found that casp-2S interacts with cytoplasmic Fodrin and inhibits its cleavage during apoptosis. As a result, casp-2S selectively inhibits the membrane blebbing and phosphatidylserine externalization that are characteristics of apoptosis.

## Materials and Methods

### Cell Culture and treatment

The human ovarian cancer cell line A2780 and its resistant subline CP70 [30] were kindly provided by Dr. Paul Modrich (Duke University). Another A2780-derivative resistant subline, CDDP [31], was kindly provided by Dr. Karuppaiyah Selvendirany (The Ohio State University). The A2780-derivative cisplatin-resistant cell lines were produced by exposing the sensitive parental line to intermittent and incrementally increasing concentrations of cisplatin [30], [31]. SKOV-3, PEO1 and PEO4 ovarian cancer cell lines were kindly provided by Dr. Thomas C. Hamilton (Fox Chase Cancer Center) and have been described previously [32]. Ovarian cancer cell line 2008 and its resistant cell line 2008C13 were kindly provided by Dr. Francois X. Claret (University of Texas - M. D. Anderson Cancer Center), and have been characterized [33], [31], [34]. A549, H460 and H1299 cell lines (originally purchased from ATCC) were kindly provided by Dr. Wenrui. Duan (The Ohio State University). HeLa cells with overexpression of casp-2S (HeLa-Casp-2S) were established in our own lab. These cell lines were maintained in RPMI 1640 medium (Life Technologies, Grand Island, NY) supplemented with 10% fetal bovine serum, 100 µg/ml streptomycin and 100 units/ml penicillin. HCT116(p53^−/−^) and HCT116(p53^+/+^) colorectal carcinoma cells were kindly provided by Dr. B. Vogelstein (Johns Hopkins University) [35], and were cultured in McCoy's 5A medium (Life Technologies) with 10% fetal bovine serum, 100 µg/ml streptomycin and 100 units/ml penicillin. The glioblastoma cell line Gli36 [36] was provided by Dr. Balveen Kaur (The Ohio State University), and T98G and U87MG (originally purchased from ATCC) were provided by Dr. Arnab Chakravarti (The Ohio State University). These cells were cultured in MEM medium supplied with 10% fetal bovine serum, 100 µg/ml streptomycin and 100 units/ml penicillin. All cells were grown at 37°C in humidified atmosphere of 5% CO_2_ in air.

For cisplatin treatment, cells were maintained in medium with the desired doses of cisplatin (Sigma, St. Louis, MO) for 1 h, washed with PBS, and incubated in fresh cisplatin-free medium for varying times post-treatment. For UV exposure, the cultures were washed with PBS, irradiated with UV at 20 J/m^2^, and then incubated for varying times. UV-C light (254 nm) was delivered from a germicidal lamp at a dose rate of 0.5 J/m^2^/s, as measured by a UVX digital radiometer connected to a UVX-31 sensor (UVP, Inc., Upland, CA).

### Plasmid and siRNA transfection

The plasmid encoding the full-length human casp-2S (p3XFLAG-CMV-14-casp-2S) has been described previously [21]. siRNA directed against *casp-2S* (5′- GAA UAC UAC UGG UAA ACU AUU -3′) (5′UTR), and a scramble non-targeting siRNA (5′- UUC UCC GAA CGU GUC ACG UdTdT -3′), were synthesized by Dharmacon Inc. (Lafayette, CO). The knockdown efficiency of casp-2S siRNA has been validated previously [21]. 100 nM siRNA was transfected into cells using Lipofectamine 2000 transfection reagent (Invitrogen) according to the manufacture's instruction.

### Apoptosis Analysis by Annexin V staining

Phosphatidylserine exposure on the outer leaflet of the plasma membrane was detected using the Annexin V-GFP apoptosis detection kit II (BD Pharmingen, San Diego, CA) according to the manufacturer's instructions. Briefly, 1 X 10^6^ cells were pelleted following treatment and washed in PBS. The cells were then resuspended in 500 µl of binding buffer, mixed with Annexin V-GFP and propidium iodide (PI) and incubated at room temperature (22°C) for 5–10 min in the dark. The Annexin V-positive cells were analyzed by flow cytometry.

### Purification of casp-2S and mass spectrometric analysis

1 X 10^8^ HeLa cells stably transfected with FLAG-tagged casp-2S (HeLa-Casp-2S cells) and mock-transfected HeLa cells were lysed in RIPA buffer (50 mM Tris-HCl, pH 7.4, 150 mM NaCl, 1 mM EDTA, 1% NP-40, protease inhibitor cocktail) for 30 min at 4°C. After centrifugation at 14,000 rpm for 10 min, supernatant was collected for immunoprecipitation (IP). Three milligrams of total proteins were incubated with anti-FLAG affinity gel (Sigma) for 2 h at 4°C. The resin was washed 4 times with lysis buffer and eluted with 400 µl of 3 X FLAG peptide (200 µg/ml final). The FLAG peptide elutes were precipitated with 10% TCA, washed twice with cold acetone, and dissolved in 30 µl of 6 M urea. The total protein was resolved in 8–16% gel and stained with Coomassie bright blue. The differential bands between HeLa and HeLa-Casp-2S samples were cut and subjected to in-gel protease digestion and analyzed by nano LC-MS/MS on a LTQ mass spectrometer (Mass Spectrometry & Proteomics Facility, Ohio State University, Columbus, OH). The resulting files were searched in the SwissProt 2011-06 database using Mascot Daemon.

### IP and immunoblotting

Whole cell lysates were prepared from HeLa-Casp-2S and CDDP cells using RIPA buffer. One milligram of total protein was incubated with 2 µg of rabbit anti-Fodrin antibody (Cell Signaling Technology, Danvers, MA) or normal rabbit IgG overnight at 4°C with continuous rotation. The immunoprecipitates were recovered following 2 h incubation with 30 µl of Protein G plus/Protein A agarose beads (Calbiochem, Gibbstown, NJ) and centrifugation. Western blot analysis was carried out to visualize the presence of Fodrin and casp-2S.

For immunoblotting analysis, whole cell lysates were prepared by boiling cell pellets for 10 min in SDS lysis buffer [2% SDS, 10% Glycerol, 62 mM Tris-HCl, pH 6.8 and a complete mini-protease inhibitor cocktail (Roche Applied Science, Indianapolis, IN)]. After protein quantification with Bio-Rad Dc Protein Assay (Bio-Rad Laboratories, Hercules, CA), equal amounts of proteins were loaded, separated on a polyacrylamide gel, and transferred to a nitrocellulose membrane. Protein bands were immuno-detected with appropriate antibodies, e.g., anti-casp-2 (551093, BD Bioscience, recognizes both long and short isoforms of casp-2), anti-Fodrin (Cell Signaling Technology, Danvers, MA), anti-FLAG (Sigma, St. Louis, MO), anti-Tubulin (Santa Cruz Biotechnology, Dallas, TX), anti-cleaved PARP and anti-cleaved caspase-3 (Cell Signaling Technologies).

### Subcellular fractionation

Cells were harvested and resuspended in 200 µl of lysis buffer (5 mM Tris-HCl, pH 7.4, 50 mM KCl, 5 mM MgCl_2_, 1 mM EGTA, protease inhibitors) and permeabilized with NP-40 (0.1%) for 10 min at 4°C. The cytosol and nuclei were separated by centrifuging the lysate at 14,000 rpm for 10 min. The nuclei pellets were further lysed in SDS lysis buffer. Each protein fraction, corresponding to an equivalent cell number, was loaded for SDS-PAGE and analyzed by immunoblotting with the indicated antibodies.

### Membrane blebbing detection

Membrane blebbing was detected by using the Apoptotic Blebs Assay Kit (Cayman Chemical, Ann Arbor, MI). Briefly, both floating and attached cells were harvested after treatment with cisplatin, washed with assay buffer, and incubated with scFv Fusion Protein Solution, followed by incubation with Fluorescein-labeled Rabbit IgG Solution. After adding the PI Solution, cells were observed under a fluorescent microscope. Dead cells stained with PI and showed red, while apoptotic blebs stained with Fluorescein-labeled Rabbit IgG, and showed green.

### Statistical Analysis

Evaluation of statistical significance for data from flow cytometry and membrane blebbing analysis was assessed using 2-Sample t-test. Differences were considered to be statistically significance at a value of *P*<0.05.

## Results

### The expression of casp-2 isoforms in various cancer cell lines

**Figure 1:**
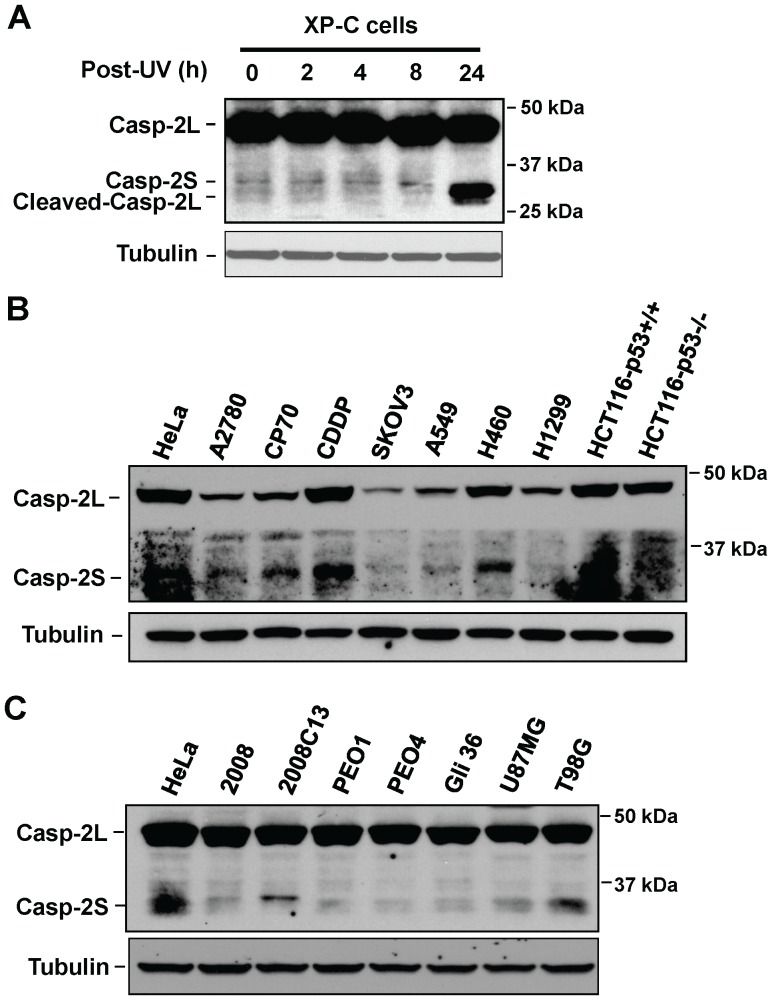
The expression of caspase-2 in various cancer cell lines. A, Human XP-C cells were UV irradiated at 10 J/m^2^, and further cultured for the indicated time periods. Whole cell lysates were prepared and subjected to Western blot to detect casp-2L, casp-2S and cleaved casp-2L simultaneously with the anti-casp-2 antibody. B, C. The expression of casp-2L and casp-2S was determined by Western blot analysis of protein extracts of the cervical cancer cell line (HeLa), ovarian cancer cell lines (A2780, CP70, CDDP, 2008, 2008C13, PEO1, PEO4 and SKOV3), lung cancer cell lines (A549, H460, and H1299), colorectal cancer cell lines (HCT116p53^+/+^ and HCT116p53^−/−^), and glioblastoma cell lines (Gli36, U87MG, and T98G) with the anti-casp-2 antibody used in A. Tubulin was detected to serve as loading control.

Although the protein expression of casp-2L has been detected in many cell lines [37], the protein level of casp-2S has seldom been revealed, and whether the casp-2S isoform exists as a protein in human cells remains a controversial issue [14]. We and others have successfully detected casp-2S in human XP-C fibroblasts and U937 monocytes [21], [38]. To further validate the specificity of this antibody and ensure the specific band detected around 33 kDa is casp-2S but not cleaved casp-2L, we UV irradiated XP-C cells and further cultured these cells for various time periods. As shown in Fig. 1A, cleaved casp-2L (30 kDa), which migrates faster than casp-2S (33 kDa), is only visible after 24 h of UV irradiation, whereas casp-2S can be clearly detected in other samples without the interference of cleaved casp-2L. Thus, the specific band around 33 kDa detected by anti-casp-2 antibody, especially in those cells without cellular stress induction, is casp-2S.

To test for the presence of casp-2S protein in human cancer cells, we determined the protein expression levels of casp-2L and casp-2S in various cancer cell lines by using this anti-casp-2 antibody [21]. As shown in Fig. 1B and C, casp-2L was detectable, with varying levels of expression, in all tested cell lines. We also detected casp-2S in various cancer cell lines, e.g., HeLa, CP70, CDDP, H460, 2008, 2008C13, PEO1 and PEO4. In contrast, casp-2S was undetectable in the SKOV3, H1299 and HCT116 p53^−/−^ cells.

It was reported that casp-2S mRNA is expressed predominantly in the heart, brain, and skeletal muscle with the highest expression found in embryonic brain [11]. However, we did not find a high casp-2S protein expression in three glioblastoma cell lines (Fig. 1C). In contrast, the highest casp-2S protein expression was found in cisplatin-resistant ovarian cancer cell lines, CDDP and 2008C13.

### Casp-2S inhibits cisplatin-induced apoptosis

**Figure 2:**
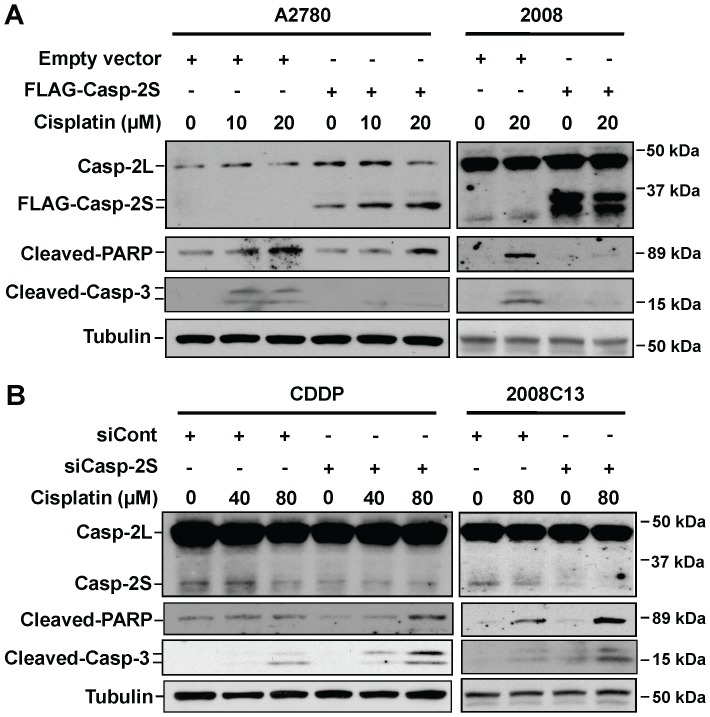
Casp-2S inhibits cisplatin-induced apoptosis. Casp-2S was either over-expressed in A2780 and 2008 cells (A), or knocked down in CDDP cells and 2008C13 cells (B) for 48 h. Cells were treated with cisplatin for another 48 h, the cleaved PARP and cleaved casp-3 were detected in these cells by using Western blotting with anti-cleaved PARP and anti-cleaved casp-3 antibodies. Meanwhile, the expression of casp-2L and casp-2S was also detected. Tubulin was blotted to serve as a loading control.

It has been reported that overexpression of casp-2S selectively prevented some phenotypic aspects of apoptosis, including chromatin condensation, apoptotic body formation and membrane blebbing in etoposide treated human leukemic U937 cells following treatment with etoposide [23]. To confirm the anti-apoptotic role of casp-2S, we either overexpressed or downregulated casp-2S in ovarian cancer cell lines and detected their effect on cisplatin-induced apoptosis. As shown in Fig. 1, A2780 and 2008 cells have an extremely low level of casp-2S, while CDDP and 2008C13 cells display a relatively high level of casp-2S. Thus, we overexpressed casp-2S in A2780 and 2008 cells (Fig. 2A), and knocked down the expression of casp-2S in CDDP and 2008C13 cells (Fig. 2B). Since there are two in-frame ATG initiation codons in casp-2S cDNA, both functioning as protein initiation sites for casp-2S synthesis [13], two bands were detected in the A2780 and 2008 cells transfected with FLAG-tagged casp-2S (Fig. 2A). Using a siRNA that only targets casp-2S [21], we were able to down-regulate the expression of casp-2S in both CDDP and 2008C13 cells without affecting casp-2L expression (Fig. 2B). Apoptosis analysis showed that over-expression of casp-2S in both A2780 and 2008 cells attenuated PARP cleavage and caspase-3 cleavage following cisplatin treatment (Fig. 2A), while down-regulation of casp-2S in CDDP and 2008C13 cells enhanced cisplatin-induced PARP cleavage and caspase-3 cleavage (Fig. 2B), indicating that casp-2S is an anti-apoptotic factor.

**Figure 3:**
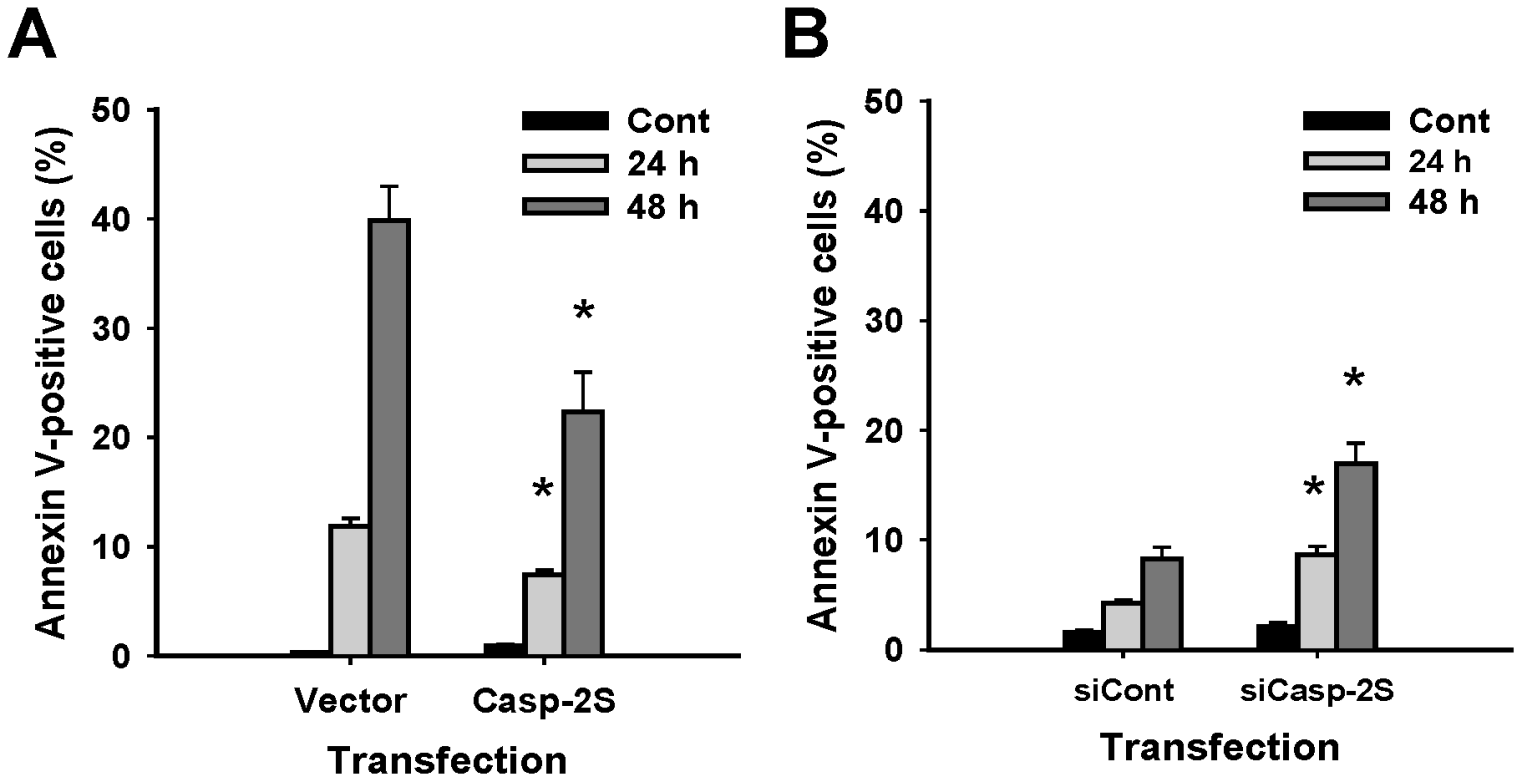
Casp-2S inhibits cisplatin-induced phosphatidylserine externalization. Casp-2S was over-expressed in A2780 cells (A), or knocked down in CDDP cells (B) for 48 h. Cells were then treated with cisplatin for another 24 and 48 h, and the phosphatidylserine externalization was detected with Annexin V staining by using Flow cytometry. n = 3, bar: SD, *: p<0.05 compared to the cisplatin-treated vector or control siRNA transfected cells at the same time point.

We also analyzed phosphatidylserine externalization in A2780 cells over-expressing casp-2S, and CDDP cells with down-regulation of casp-2S following cisplatin treatment. Similar to the etoposide-treated U937 cells [23], over-expression of casp-2S also reduced phosphatidylserine externalization in A2780 cells following cisplatin treatment, as reflected by a decrease of Annexin V-positive cells (Fig. 3A). In addition, knockdown of casp-2S in CDDP cells enhanced cisplatin-induced phosphatidylserine externalization (Fig. 3B), further confirming the inhibitory effect of casp-2S on apoptosis.

### Casp-2S interacts with cytoskeleton proteins

**Figure 4:**
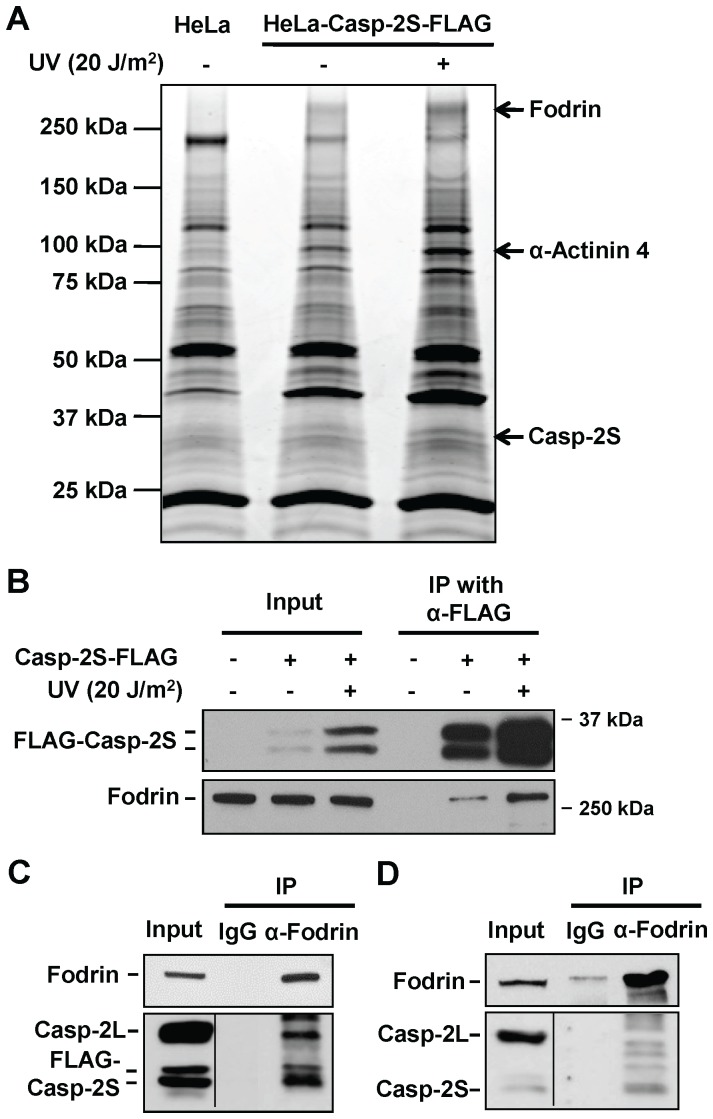
Casp-2S interacts with cytoskeleton proteins. A, Casp-2S was purified from HeLa cells stably transfected with the FLAG-tagged casp-2S using an anti-FLAG affinity gel. Purified proteins were separated by SDS-PAGE and stained with Coomassie blue. The differentially expressed bands in the HeLa-casp-2S cells were identified by mass-spectrometry. B, FLAG-tagged casp-2S was immunoprecipitated from the HeLa-Casp-2S cells and Fodrin was detected using Western blotting with the anti-Fodrin antibody. C, D, Fodrin was immunoprecipitated from the HeLa-Casp-2S cells (C) or CDDP cells (D) with an anti-Fodrin antibody, and the existence of casp-2S was detected with anti-casp-2S antibody.

To explore the molecular roles of casp-2S in cells, we purified casp-2S as multimeric complexes and analyzed the co-purified proteins using mass spectrometry. Casp-2S was stably expressed as FLAG-epitope fusions in HeLa cells. FLAG-tagged casp-2S was purified from whole cell lysates using IP with anti-FLAG affinity gel. The purified complexes were further separated on an 8–16% SDS-PAGE gel. As a control, we performed a mock purification from untransfected HeLa cells. Coomassie blue staining of the protein bands is shown in Fig. 4A. We found three differentially displayed bands in the HeLa-Casp-2S cells in comparison to the untransfected HeLa cells. Using mass spectrometric analysis, Fodrin was detected in the 270 kDa band, α-Actinin 4 was detected in the 100 kDa band, and casp-2S was detected in 36 kDa band. We further confirmed the interaction between casp-2S and Fodrin through use of reciprocal IP and immunoblotting. As shown in Fig. 4B and 4C, Fodrin was detectable in the complex immunoprecipitated with the anti-FLAG antibody, and FLAG-tagged casp-2S was detectable in the complex immunoprecipitated with the anti-Fodrin antibody in HeLa-Casp-2S-FLAG cells. We further attempted to investigate the interaction between Fodrin and endogenous casp-2S in CDDP cells. As shown in Fig. 4D, the anti-Fodrin antibody is able to pull down endogenous casp-2S but not casp-2L. Thus, our data suggests that casp-2S forms a complex with cytoskeleton proteins.

### Casp-2S inhibits cytoplasmic Fodrin cleavage during apoptosis

**Figure 5:**
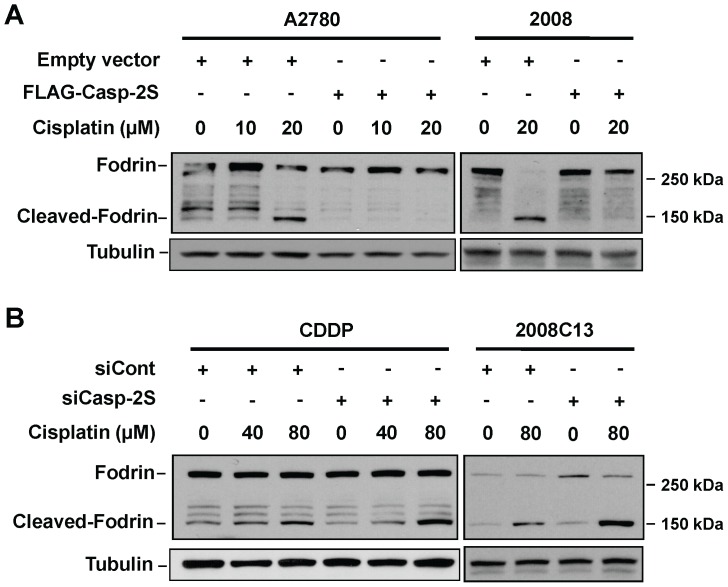
Casp-2S inhibits cisplatin-induced Fodrin cleavage. Casp-2S was either over-expressed in A2780 cells and 2008 cells (A), or knocked down in CDDP cells and 2008C13 cells (B) for 48 h as in Fig. 2. Cells were treated with cisplatin for another 48 h, and the cleavage of Fodrin was detected with the anti-Fodrin antibody. Tubulin was detected as a loading control.

**Figure 6:**
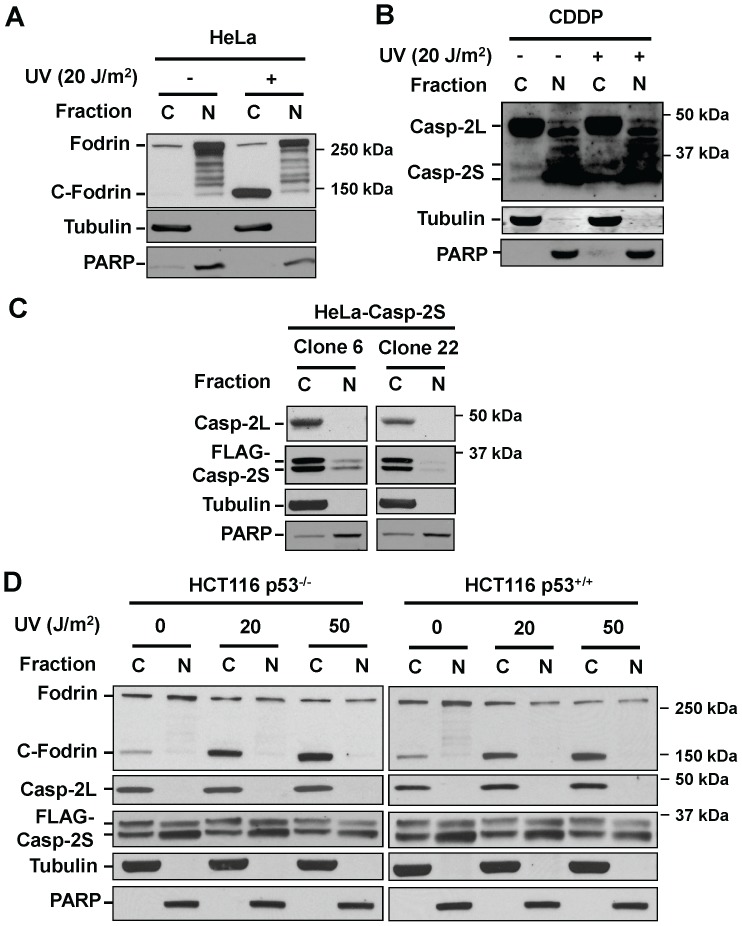
Casp-2S and cleavage of Fodrin mainly occur in cytoplasm. A, HeLa cells were UV irradiated at 20 J/m^2^, and further cultured for 4 h. Cytoplasm (C) and nuclei (N) were separated and the Fodrin cleavage in protein extracts was detected with an anti-Fodrin antibody. Tubulin and PARP were used to indicate the cytoplasm and nuclei fraction, respectively. B. CDDP cells were fractionated into cytoplasm and nuclei, the endogenous casp-2L and casp-2S were detected simultaneously with the anti-casp-2 antibody. C, Two clones of casp-2S stably expressed HeLa cells were fractionated to the cytoplasm and nuclei, casp-2L and FLAG-tagged casp-2S were detected with the anti-casp-2 and the anti-FLAG antibodies, respectively. D, HCT116 p53^−/−^ and HCT116 p53^+/+^ cells were transiently transfected with FLAG-tagged casp-2S, UV irradiated at various doses, and further cultured for 4 h. The cytoplasm and nuclei were separated, the Fodrin cleavage, casp-2L, and FLAG-tagged casp-2S were detected with anti-Fodrin, anti-casp-2 and anti-FLAG antibodies, respectively.

The cleavage of Fodrin is an early event in apoptosis [28]. Since we have found that casp-2S interacts with Fodrin, we sought to know whether this interaction protects Fodrin from being cleaved during apoptosis. Again, FLAG-tagged casp-2S was over-expressed in A2780 and 2008 cells, and the endogenous casp-2S in CDDP and 2008C13 cells was specifically knocked down. These cells were treated with cisplatin, and the cleavage of Fodrin was determined using immunoblotting with the anti-Fodrin antibody. As shown in Fig. 5, when the cells were treated with cisplatin, Fodrin cleavage was reduced in the A2780 and 2008 cells over-expressing casp-2S (Fig. 5A), while cleavage was enhanced in CDDP and 2008C13 cells with casp-2S down-regulation (Fig. 5B). Given casp-2 and Fodrin are present in both the cytosol and nucleus [39], [40], [41], we determined the location where Fodrin is cleaved during apoptosis. HeLa cells were UV irradiated to induce apoptosis, and fractionated into the cytosolic and nuclear components. Both the existence and cleavage of Fodrin were detected in these two fractions. The results indicate that UV-induced Fodrin cleavage occurs exclusively within the cytoplasm (Fig. 6A).

We then attempted to detect the subcellular localization of endogenous casp-2L and casp-2S in CDDP cells. The cytoplasm and nucleus were fractionated, and the presence of casp-2L and casp-2S were detected simultaneously with the anti-casp-2 antibody. As shown in Fig. 6B, both casp-2L and casp-2S can be detected in the cytoplasm and nucleus with casp-2L predominantly residing in the cytoplasm while casp-2S mainly localizing in the nucleus. UV irradiation did not change the localization of casp-2L, but increased the amount of cytoplasmic casp-2S.

To easily detect the subcellular localization of casp-2S, HeLa cells were stably transfected with FLAG-tagged casp-2S. The cytoplasm and nucleus were fractionated in two clones of FLAG-tagged casp-2S over-expressed HeLa cells. The presence of casp-2L and casp-2S were detected with anti-casp-2 and anti-FLAG antibodies, respectively. As shown in Fig. 6C, casp-2L was detected exclusively in the cytosol, but not in the nucleus. Ectopically expressed casp-2S, however, was detectable in both the cytosol and nucleus, and found to exist predominantly in the cytosol. We then analyzed the subcellular distribution of casp-2L and ectopically expressed FLAG-tagged casp-2S, as well as cleavage of Fodrin in HCT116 cells with different p53 status. As shown in Fig. 6D, UV-induced Fodrin cleavage occurs exclusively in the cytoplasm and is independent of p53 function. Again, casp-2L was only detected in the cytoplasm, but not in the nucleus, whereas transiently transfected FLAG-tagged casp-2S was equally detected in both the cytoplasm and nucleus. In addition, the distribution of casp-2L and FLAG-tagged casp-2S was not affected by the status of p53. This data indicates that the subcellular localization of casp-2L and casp-2S is cell type-dependent, and it is the cytoplasmic casp-2S that protects Fodrin cleavage during apoptosis.

### Casp-2S inhibits apoptotic blebbing

**Figure 7:**
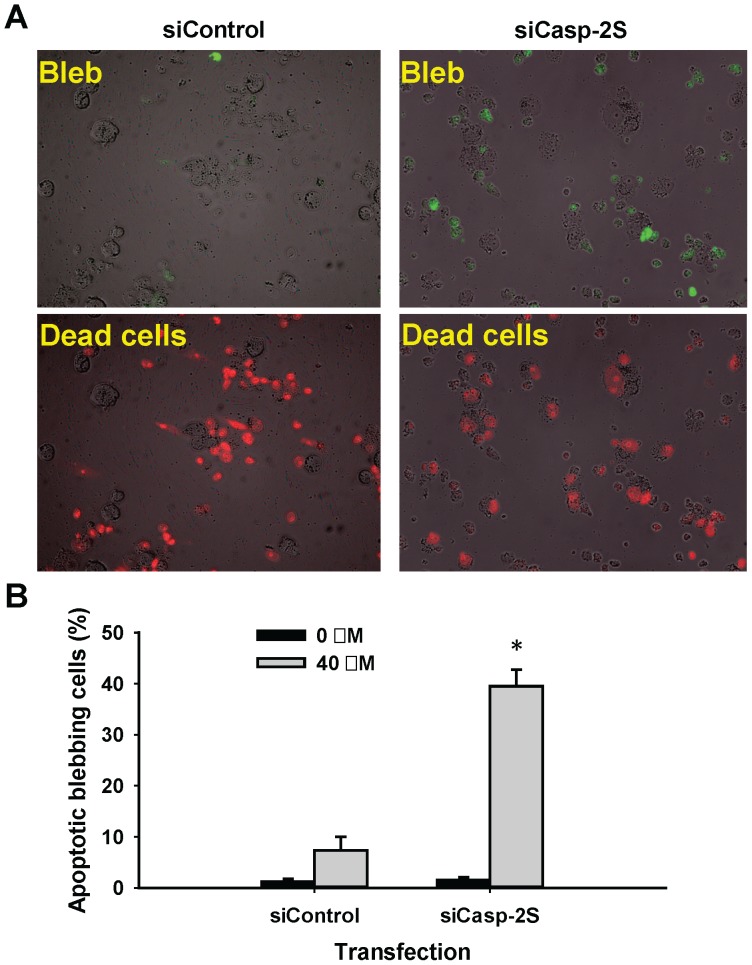
Caps-2S inhibits cisplatin-induced membrane blebbing. A, CDDP cells were transfected with casp-2S siRNA or control siRNA for 48 h, and treated with cisplatin for 48 h. The apoptotic bleb and dead cells were stained separately as described in Materials and Methods. B, The number of apoptotic blebbing cells were counted and plotted. n = 50, bar: SD, *: p<0.05 compared to siControl.

The Fodrin-actin scaffold underlying the lipid bilayer is considered to participate in cell-shape stabilization. Proteolysis of Fodrin, which occurs during apoptosis, leads to the destabilization of the scaffold, and produces membrane blebbing [28]. It has been reported that casp-2S over-expression prevents membrane blebbing in human leukemia cell line U937 and human B lymphoma Namalwa cells following VP-16 treatment [23], [25]. To further evaluate the effect of casp-2S on apoptotic blebbing under physiological conditions, we used casp-2S siRNA to knock down casp-2S expression in the human ovarian cancer cell line CDDP, which expresses a high level of casp-2S, and analyzed membrane blebbing after cisplatin treatment. We found that down-regulation of casp-2S significantly increased apoptotic blebbing in CDDP cells (Fig. 7A, B). Given our finding that casp-2S prevents Fodrin cleavage during apoptosis, we reasoned that casp-2S inhibits apoptotic blebbing through stabilization of the Fodrin-related cytoskeleton scaffold.

## Discussion

A major contributing factor for the confounding results describing the role of casp-2 in apoptosis is the existence and differential expression of casp-2L and casp-2S, which exhibit opposing functions in apoptosis [11]. Casp-2L (always referred to as casp-2) mRNA expression was observed to be dominant over casp-2S. This is most likely due to a stronger promoter [13] and the instability of casp-2S mRNA [14]. The casp-2L protein was abundantly expressed in numerous human and mouse cell lines [37]. The protein expression of casp-2S, in contrast, was seldom detected because its predicted mass is very close to that of the intermediate cleavage product of casp-2L. Furthermore, the casp-2S mRNA was shown to be very short-lived and thus may normally be expressed at very low levels. Nevertheless, we have successfully detected the casp-2S protein in human XP-C cells, as well as in various cancer cell lines in this study, confirming that casp-2S protein does exist in cells and its expression level depends on cell type.

One remarkable feature of casp-2 is its nuclear localization. Different from all other caspases, casp-2 has been widely detected within the nucleus [37], [42], [40], [43], [44], [45], [46], [47]. However, the subcellular localization of casp-2 continues to be debated. Although overwhelming evidence demonstrates the predominant casp-2 localization in the nucleus, this has been contradicted by others showing that the majority of casp-2 is detected in the cytoplasm by using both fractionation and microscopic analysis [44], [39], [48], [49]. Thus, further studies are necessary to unambiguously address this issue, especially the distinct localization of casp-2L and casp-2S. Our ability to successfully detect the individual isoforms of casp-2 protein expression enabled us to detect the subcellular localization of both casp-2L and casp-2S. Our subcellular fractionation analysis revealed that casp-2L was predominantly detected in the cytosol with a relatively small amount in nucleus. Casp-2S, however, was detected in both the cytosol and nucleus, with the predominant subcellular localization of casp-2S being dependent on the cell type. It was proposed that the accumulation of casp-2 (primarily casp-2L) in the cytosolic compartment observed after subcellular fractionation might be attributed to the dissociation of casp-2 from the nucleus as a result of the experimental procedure [50]. Although we failed to detect the existence of casp-2L in the nucleus of HeLa and HCT116 cells, we did detect casp-2S and Fodrin in the nuclear compartment of the same sample. This indicates that the absence of nuclear casp-2L in HeLa and HCT116 cells does not stem from an error in the fractionation procedure. Another speculation is that redistribution of nuclear casp-2 may occur upon cell lysis; pre-treatment of cells with N-ethylmaleimide (NEM) for 10 min increased the amount of casp-2 in the nuclear fraction, suggesting that NEM treatment can completely prevent redistribution of casp-2 from the nucleus to the extra-nuclear fraction when cells are lysed [51]. However, it is also possible that NEM treatment itself can induce the translocation of casp-2 to the nucleus. In addition, many immunofluorescence analyses, which do not lyse the cell, also support the predominant cytosolic localization of casp-2 [39], [48]. Thus, this apparent discrepancy in the subcellular localization of casp-2 is likely due to differences among different cell types.

Casp-2S has been shown to selectively inhibit apoptotic-blebbing and -body formation during apoptosis. It is well known that membrane blebbing and apoptotic body formation require important remodeling of the cytoskeleton [52], [53], which is normally maintained by the Spectrin-Actin scaffold. Fodrin is a major substrate for proteases during apoptosis. Proteolysis of Fodrin leads to the destabilization of the Spectrin network and the loss of Actin filament crosslinks [28], [54], [55], which is thought to be involved in apoptotic membrane blebbing [28]. During apoptosis, casp-3 appears to be the major executioner caspase of Spectrin [56]. However, several lines of evidence suggest a casp-3 independent cleavage [57], [58], and that Fodrin can be cleaved by casp-2, casp-7 and calpain. It has been reported that Fodrin cleavage by casp-2, 3 and 7 is inhibited by the binding of calmodulin to Fodrin [59]. By using ectopic expression of FLAG-tagged casp-2S and mass spectrometric analysis, we revealed that the cytoskeleton protein Fodrin and α-actinin 4 bind to casp-2S under the physiological conditions, and this binding protects the cleavage of Fodrin and prevents the formation of membrane blebbing during apoptosis. In addition, our data also showed that casp-2L interacts with Fodrin in casp-2S overexpressing cells. Thus, it is also possible that casp-2S may bind to and inhibit casp-2L-mediated cleavage of Fodrin and apoptosis.

There is no doubt that apoptosis has dramatic effects on the cytoskeleton. However, disruption of the cytoskeleton by itself can induce or accelerate apoptosis [60], [61], [62]. Given the role of casp-2S in protecting cytoskeleton protein Fodrin from being cleaved, it is also possible that casp-2S may inhibit cytoskeleton damage induced apoptosis.

In non-erythroid cells, Fodrin is not only distributed at the plasma membrane but is also present in the nucleus [41]. Our subcellular fractionation analysis clearly demonstrated that the cleavage of Fodrin during apoptosis occurs exclusively in the cytoplasm. Thus, it is the cytosolic casp-2S, not the nuclear casp-2S, which interacts with Fodrin and protects its cleavage. While not involved in protection of Fodrin cleavage, the function of nuclear casp-2S is still unclear. It has been suggested that nuclear casp-2 might play a role in tumor suppression by functioning in DNA damage signaling [63]. However, whether this role is mediated by casp-2L or casp-2S is unknown and warrants further study.

Casp-2S has been shown to interact with various proteins. By using yeast two-hybrid screening, ISBP was identified as possessing an interaction with casp-2S. This interaction has been proposed as a means to mediate the anti-apoptotic function of casp-2S by preventing procaspase-2L processing and activation [24]. Casp-2S was also found to interact with caspase-3, inhibiting procaspase-3 processing and activation, thus preventing ROCK-1 activation [25]. Activated ROCK-1 induces membrane blebbing through enhanced myosin light chain phosphorylation, actin filamentous structures stabilization, and promotion of actin-myosin contractile force [64]. Casp-2S-mediated ROCK-1 inactivation was proposed to contribute to the inhibition of apoptotic blebbing by casp-2S. Our finding that casp-2S binds to Fodrin and inhibits its cleavage provides another mechanism underlying the anti-apoptotic function of casp-2S, and further supports the survival role of casp-2S.

In summary, we have identified a novel mechanism for the inhibition of apoptosis and apoptotic bleb formation by casp-2S. Given casp-2S executes its inhibitory role in preventing Fodrin cleavage in the cytoplasm, the function of nucleus-localized casp-2S remains to be elucidated.
